# A multilayered post-GWAS assessment on genetic susceptibility to pancreatic cancer

**DOI:** 10.1186/s13073-020-00816-4

**Published:** 2021-02-01

**Authors:** Evangelina López de Maturana, Juan Antonio Rodríguez, Lola Alonso, Oscar Lao, Esther Molina-Montes, Isabel Adoración Martín-Antoniano, Paulina Gómez-Rubio, Rita Lawlor, Alfredo Carrato, Manuel Hidalgo, Mar Iglesias, Xavier Molero, Matthias Löhr, Christopher Michalski, José Perea, Michael O’Rorke, Victor Manuel Barberà, Adonina Tardón, Antoni Farré, Luís Muñoz-Bellvís, Tanja Crnogorac-Jurcevic, Enrique Domínguez-Muñoz, Thomas Gress, William Greenhalf, Linda Sharp, Luís Arnes, Lluís Cecchini, Joaquim Balsells, Eithne Costello, Lucas Ilzarbe, Jörg Kleeff, Bo Kong, Mirari Márquez, Josefina Mora, Damian O’Driscoll, Aldo Scarpa, Weimin Ye, Jingru Yu, PanGenEU Investigators, Montserrat García-Closas, Manolis Kogevinas, Nathaniel Rothman, Debra T Silverman, SBC/EPICURO Investigators, Demetrius Albanes, Alan A Arslan, Laura Beane-Freeman, Paige M Bracci, Paul Brennan, Bas Bueno-de-Mesquita, Julie Buring, Federico Canzian, Margaret Du, Steve Gallinger, J Michael Gaziano, Phyllis J Goodman, Marc Gunter, Loic LeMarchand, Donghui Li, Rachael E Neale, Ulrika Peters, Gloria M Petersen, Harvey A Risch, Maria José Sánchez, Xiao-Ou Shu, Mark D Thornquist, Kala Visvanathan, Wei Zheng, Stephen J Chanock, Douglas Easton, Brian M Wolpin, Rachael Z Stolzenberg-Solomon, Alison P Klein, Laufey T Amundadottir, Marc A Marti-Renom, Francisco X Real, Núria Malats

**Affiliations:** 1 Genetic and Molecular Epidemiology Group, Spanish National Cancer Research Center (CNIO), C/Melchor Fernandez Almagro 3, 28029 Madrid, Spain; 2 CIBERONC, Madrid, Spain; 3 CNAG-CRG, Centre for Genomic Regulation (CRG), Barcelona Institute of Science and Technology (BIST), Barcelona, Spain; 4 ARC-Net Centre for Applied Research on Cancer and Department of Pathology and Diagnostics, University and Hospital Trust of Verona, Verona, Italy; 5 Department of Oncology, Ramón y Cajal University Hospital, IRYCIS, Alcala University, Madrid, Spain; 6 Madrid-Norte-Sanchinarro Hospital, Madrid, Spain; 7 Weill Cornell Medicine, New York, USA; 8 Hospital del Mar—Parc de Salut Mar, Barcelona, Spain; 9 Hospital Universitari Vall d’Hebron, Vall d’Hebron Research Institute (VHIR), Barcelona, Spain; 10 Universitat Autònoma de Barcelona and CIBEREHD, Barcelona, Spain; 11 Gastrocentrum, Karolinska Institutet and University Hospital, Stockholm, Sweden; 12 Department of Surgery, Technical University of Munich, Munich, Germany; 13 Department of Visceral, Vascular and Endocrine Surgery, Martin-Luther-University Halle-WittenberHalle (Saale), Halle, Germany; 14 Department of Surgery, Hospital 12 de Octubre, and Department of Surgery and Health Research Institute, Fundación Jiménez Díaz, Madrid, Spain; 15 Centre for Public Health, Queen’s University Belfast, Belfast, UK; 16 College of Public Health, The University of Iowa, Iowa City, IA USA; 17 Molecular Genetics Laboratory, General University Hospital of Elche, Elche, Spain; 18 Department of Medicine, Instituto Universitario de Oncología del Principado de Asturias (IUOPA), Instituto de Investigación Sanitaria del Principado de Asturias (ISPA), Oviedo, Spain; 19 CIBERESP, Madrid, Spain; 20 Department of Gastroenterology and Clinical Biochemistry, Hospital de la Santa Creu i Sant Pau, Barcelona, Spain; 21 Department of Surgery, Hospital Universitario de Salamanca – IBSAL, Universidad de Salamanca, Salamanca, Spain; 22 Barts Cancer Institute, Centre for Molecular Oncology, Queen Mary University of London, London, UK; 23 Department of Gastroenterology, University Clinical Hospital of Santiago de Compostela, Santiago de Compostela, Spain; 24 Department of Gastroenterology, University Hospital of Giessen and Marburg, Marburg, Germany; 25 Department of Molecular and Clinical Cancer Medicine, University of Liverpool, Liverpool, UK; 26 National Cancer Registry Ireland and HRB Clinical Research Facility, University College Cork, Cork, Ireland; 27 Newcastle University, Institute of Health & Society, Newcastle, UK; 28 Centre for Stem Cell Research and Developmental Biology, University of Copenhagen, Copenhagen, Denmark; 29 Department of Genetics and Development, Columbia University Medical Center, New York, NY USA; 30 Department of Systems Biology, Columbia University Medical Center, New York, NY USA; 31 Department of Medical Epidemiology and Biostatistics, Karolinska Institutet, Stokholm, Sweden; 32 Division of Cancer Epidemiology and Genetics, National Cancer Institute, National Institutes of Health, Bethesda, MD USA; 33 Institut Municipal d’Investigació Mèdica – Hospital del Mar, Centre de Recerca en Epidemiologia Ambiental (CREAL), Barcelona, Spain; 34 Department of Obstetrics and Gynecology, New York University School of Medicine, New York, NY USA; 35 Department of Environmental Medicine, New York University School of Medicine, New York, NY USA; 36 Department of Population Health, New York University School of Medicine, New York, NY USA; 37 Department of Epidemiology and Biostatistics, University of California, San Francisco, CA USA; 38 International Agency for Research on Cancer (IARC), Lyon, France; 39 Deparment for Determinants of Chronic Diseases (DCD), National Institute for Public Health and the Environment (RIVM), Bilthoven, The Netherlands; 40 Division of Preventive Medicine, Brigham and Women’s Hospital, Boston, MA USA; 41 Genomic Epidemiology Group, German Cancer Research Center (DKFZ, Heidelberg, Germany; 42 Department of Epidemiology and Biostatistics, Memorial Sloan Kettering Cancer Center, New York, NY USA; 43 Prosserman Centre for Population Health Research, Lunenfeld-Tanenbaum Research Institute, Sinai Health System, Toronto, ON Canada; 44 Departments of Medicine, Brigham and Women’s Hospital, VA Boston and Harvard Medical School, Boston, MA USA; 45 SWOG Statistical Center, Fred Hutchinson Cancer Research Center, Seattle, WA USA; 46 Cancer Epidemiology Program, University of Hawaii Cancer Center, Honolulu, HI USA; 47 University of Texas MD Anderson Cancer Center, Houston, TX USA; 48 Population Health Department, QIMR Berghofer Medical Research Institute, Brisbane, Queensland Australia; 49 Division of Public Health Sciences, Fred Hutchinson Cancer Research Center, Seattle, WA USA; 50 Department of Health Sciences Research, Mayo Clinic College of Medicine, Rochester, MN USA; 51 Department of Chronic Disease Epidemiology, Yale School of Public Health, New Haven, CT USA; 52 Escuela Andaluza de Salud Pública (EASP), Granada, Spain; 53 Instituto de Investigación Biosanitaria Granada, Granada, Spain; 54 Centro de Investigación Biomédica en Red de Epidemiología y Salud Pública (CIBERESP), Madrid, Spain; 55 Universidad de Granada, Granada, Spain; 56 Division of Epidemiology, Department of Medicine, Vanderbilt Epidemiology Center, Vanderbilt-Ingram Cancer Center, Vanderbilt University School of Medicine, Nashville, TN USA; 57 Centre for Cancer Genetic Epidemiology, Department of Public Health and Primary Care, University of Cambridge, Cambridge, UK; 58 Department Medical Oncology, Dana-Farber Cancer Institute, Boston, USA; 59 Department of Oncology, Sidney Kimmel Comprehensive Cancer Center, Johns Hopkins School of Medicine, Baltimore, MD USA; 60 Laboratory of Translational Genomics, Division of Cancer Epidemiology and Genetics, National Cancer Institute, National Institutes of Health, Bethesda, MD USA; 61 National Centre for Genomic Analysis (CNAG), Centre for Genomic Regulation (CRG), Barcelona Institute of Science and Technology (BIST), Universitat Pompeu Fabra (UPF), ICREA, Baldiri Reixac 4, 08028 Barcelona, Spain; 62 Epithelial Carcinogenesis Group, Spanish National Cancer Research Center (CNIO), Madrid, Spain; 63 Departament de Ciències Experimentals i de la Salut, Universitat Pompeu Fabra, Barcelona, Spain

**Keywords:** Pancreatic cancer risk, Genome-wide association analysis, Genetic susceptibility, 3D genomic structure, Local indices of genome spatial autocorrelation

## Abstract

**Background:**

Pancreatic cancer (PC) is a complex disease in which both non-genetic and genetic factors interplay. To date, 40 GWAS hits have been associated with PC risk in individuals of European descent, explaining 4.1% of the phenotypic variance.

**Methods:**

We complemented a new conventional PC GWAS (1D) with genome spatial autocorrelation analysis (2D) permitting to prioritize low frequency variants not detected by GWAS. These were further expanded via Hi-C map (3D) interactions to gain additional insight into the inherited basis of PC. In silico functional analysis of public genomic information allowed prioritization of potentially relevant candidate variants.

**Results:**

We identified several new variants located in genes for which there is experimental evidence of their implication in the biology and function of pancreatic acinar cells. Among them is a novel independent variant in *NR5A2* (rs3790840) with a meta-analysis *p* value = 5.91E−06 in 1D approach and a Local Moran’s Index (LMI) = 7.76 in 2D approach. We also identified a multi-hit region in *CASC8*—a lncRNA associated with pancreatic carcinogenesis—with a lowest *p* value = 6.91E−05. Importantly, two new PC loci were identified both by 2D and 3D approaches: *SIAH3* (LMI = 18.24), *CTRB2/BCAR1* (LMI = 6.03), in addition to a chromatin interacting region in *XBP1*—a major regulator of the ER stress and unfolded protein responses in acinar cells—identified by 3D; all of them with a strong in silico functional support.

**Conclusions:**

This multi-step strategy, combined with an in-depth in silico functional analysis, offers a comprehensive approach to advance the study of PC genetic susceptibility and could be applied to other diseases.

**Supplementary Information:**

The online version contains supplementary material available at 10.1186/s13073-020-00816-4.

## Background

Pancreatic cancer (PC) has a relatively low incidence, but it is one of the deadliest tumors. In Western countries, PC ranks fourth among cancer-related deaths with 5-year survival of 3–7% in Europe [1–3]. In the last decades, progress in the management of patients with PC has been meager. In addition, mortality is rising [2] and it is estimated that PC will become the second cause of cancer-related deaths in the USA by 2030 [4].

PC is a complex disease in which both genetic and non-genetic factors participate. However, relatively little is known about its etiologic and genetic susceptibility background. In comparison with other major cancers, fewer genome-wide association studies (GWAS) have been carried out and the number of patients included in them is relatively small (*N* = 9040). According to the GWAS Catalog (January 2019) [5], 40 common germline variants sited in 32 loci and associated with PC risk have been identified in individuals of European descent [6–11]. However, these variants only explain 4.1% of the phenotypic variance for PC [12]. More importantly, given the challenges in performing new PC case-control studies with adequate clinical, epidemiological, and genetic information, the field is far from reaching the statistical power that has been achieved in other more common cancers such as breast, colorectal, or prostate cancers with > 100,000 subjects included in GWAS, yielding a much larger number of genetic variants associated with them [5].

Current GWAS methodology relies on establishing simple SNP-disease associations by setting a strict statistical threshold of significance (*p* value = 5 × 10^−8^) and replicating them in independent studies. This approach has been successful in minimizing false positive hits at the expense of discarding variants that may be truly associated with the disease displaying association *p* values not reaching genome-wide significance after multiple testing correction or not being replicated in independent populations. The false negatives can be the result of weak associations or of low prevalence of the variant SNP assessed, among others. The “simple” solution to this problem is to increase the sample size. However, it will take considerable time for PC GWAS to reach the number of subjects achieved in other tumors and the funding climate for replication studies is extremely weak. While a meta-analysis based on available datasets provides an alternative strategy for novel variant identification, this approach introduces heterogeneity because studies differ regarding methods, data quality, testing strategies, genetic background of the included individuals (e.g., population substructure), and study design, factors that can lead to lack of replicability. Therefore, we are faced with the need of exploring alternative approaches to discover new putative genetic risk variants missed by conventional GWAS criteria.

Here, we build upon one of the largest PC case-control studies with extensive standardized clinical and epidemiological data annotation and apply both a classical GWAS approach (1D strategy) and novel strategies for risk-variant discovery. We use, for the first time in genomics, the Local Moran’s Index (LMI) [13], an approach that is widely followed in geospatial statistics. In its original application to geographic two-dimensional (2D) analysis, LMI identifies the existence of relevant autocorrelated clusters in the spatial arrangement of a variable, highlighting points closely surrounded by others with similar risk estimate values, allowing the identification of “hot spots.” We computed LMI of (genomic) spatial autocorrelation to identify clusters of SNPs based on their similar risk estimates (odds ratio, OR) weighted by their genomic distance as measured by linkage disequilibrium (LD). By capturing LD structures of nearby SNPs, LMI leverages the values of SNPs with low minor allele frequencies (MAFs) that conventional GWAS fail to assess properly. In this regard, LMI offers a novel opportunity to identify potentially relevant new sets of genomic candidates associated with PC genetic susceptibility.

In addition, we have taken advantage of recent advances in 3D genomic analyses providing insights into the spatial relationship of regulatory elements and their target genes. Since GWAS have largely identified variants present in non-coding regions of the genome, a challenge has been to ascribe such variants to the corresponding regulated genes, which may lie far away in the genomic sequence. Chromosome Conformation Capture experiments (3C-related techniques) [14] can provide insight into the biology and function underlying previously “unexplained” hits, in addition to identify further genetic susceptibility loci [15, 16].

**Fig. 1:**
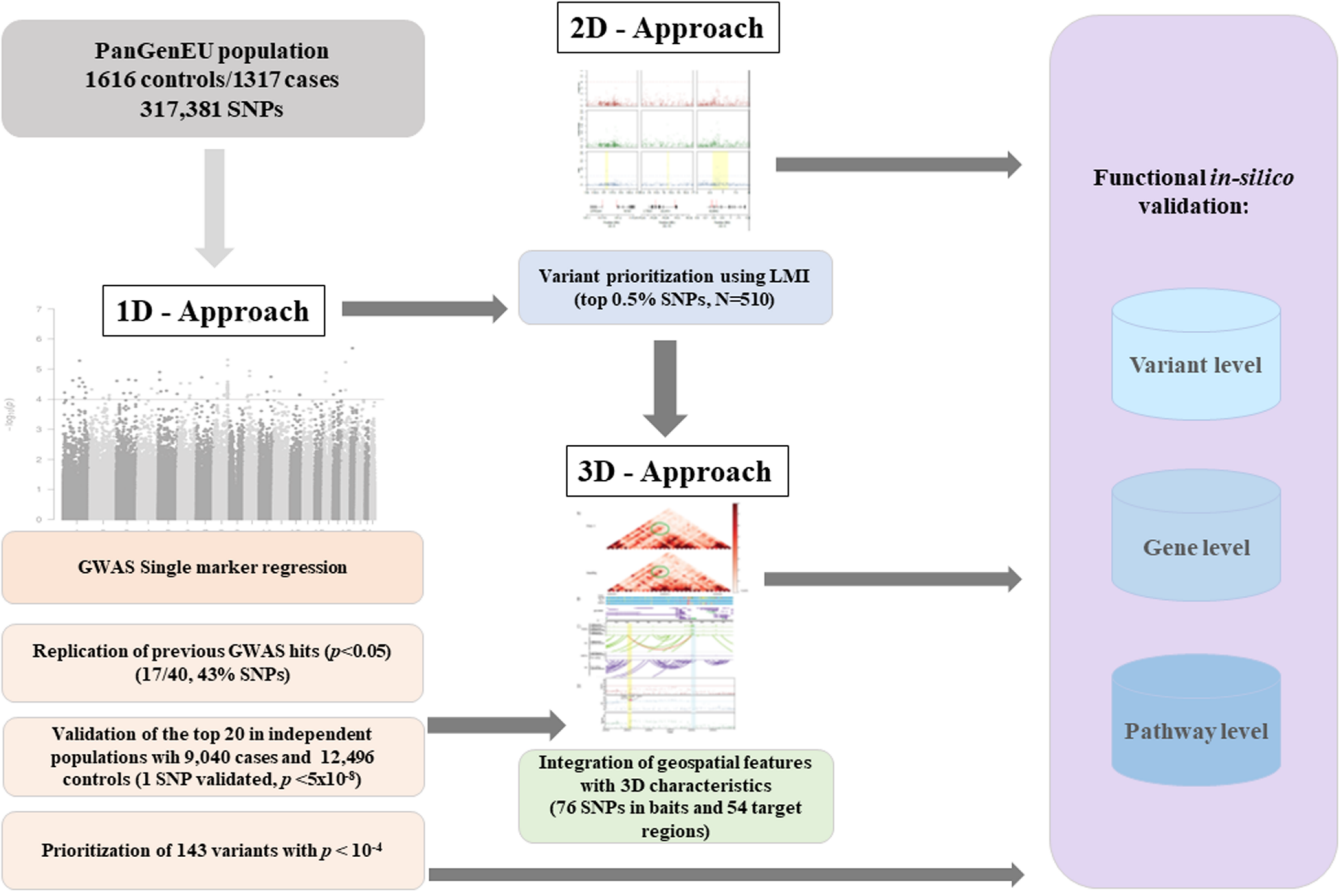
Study flowchart: overview of the complementary approaches adopted in this study to identify new pancreatic cancer susceptibility regions

The combined use of conventional GWAS (1D) analysis with LMI (2D) and 3D genomic approaches has allowed enhancing the discovery of novel candidate variants involved in PC genetic susceptibility (Fig. 1). As high-throughput technologies have produced large amounts of publicly available data from cell types and tissues, these resources represent a valuable approach to perform an in silico functional validation of prioritized variants using novel criteria, as well as for functional interpretation of genetic findings. Importantly, here we identified several new variants located in genes for which there is functional evidence of their implication in the biology of pancreatic acinar cells. Among them are a novel independent variant in *NR5A2*, a multi-hit region in *CASC8*, and three new PC loci in *SIAH3*, *CTRB2/BCAR1*, and *XBP1*, all of them with strong in silico functional support.

## Methods

### 1D approach: PanGenEU GWAS—single marker association analyses

#### Study population

We used the resources from the PanGenEU case-control study conducted in Spain, Italy, Sweden, Germany, UK, and Ireland, between 2009 and 2014 [17, 18]. Eligible PC patients, men and women ≥ 18 years of age, were invited to participate. Eligible controls were hospital in-patients with primary diagnoses not associated with known risk factors of PC. Controls from Ireland and Sweden were population-based. Institutional Review Board approval and written informed consent were obtained from all participating centers and study participants, respectively. To increase statistical power, we included controls from the Spanish Bladder Cancer (SBC)/EPICURO study, carried out in the same geographical areas where PanGenEU study was conducted. Characteristics of the study populations are detailed in Additional file 1: Table S1.

#### Genotyping and quality control of PanGenEU study subjects

DNA samples were genotyped using the Infinium OncoArray-500K [19] at the CEGEN facility (Spanish National Cancer Research Center, CNIO, Madrid, Spain). Genotypes were called using the GenTrain 2.0 cluster algorithm in GenomeStudio software v.2011.1.0.24550 (Illumina, San Diego, CA). Genotyping quality control criteria considered the missing call rate, unexpected heterozygosity, discordance between reported and genotyped gender, unexpected relatedness, and estimated European ancestry < 80%. After removing samples that did not pass the quality control filters, duplicated samples, and individuals with incomplete data regarding age of diagnosis/recruitment, 1317 cases and 700 controls were available for the association analyses. SNPs in sex chromosomes and those that did not pass the Hardy-Weinberg equilibrium (*p* value < 10^−6^) were also discarded. Overall, 451,883 SNPs passed the quality control filters conducted before the imputation.

#### Genotyping and quality control of SBC/EPICURO controls

Genotyping of germline DNA was performed using the Illumina 1M Infinium array at the National Cancer Institute (NCI) Core Genotyping Facility as previously described [20], which provided calls for 1,072,820 SNP genotypes. We excluded SNPs in sex chromosomes, those with a low genotyping rate (< 95%), and those that did not pass the Hardy-Weinberg equilibrium threshold. In addition, the exome of 36 controls was sequenced with the TruSeq DNA Exome and a standard quality control procedure both at the SNP and individual level was applied: SNPs with read depth < 10 and those that did not pass the tests of base sequencing quality, strand bias or tail distance bias, were considered as missing and imputed (see the “Imputation” section for further details). Overall, 1,122,335 SNPs were available for imputation from a total of 916 additional controls.

#### Imputation

Imputation was performed at the Wellcome Sanger Institute (Cambridge, UK) and at CNIO for the PanGenEU and the SBC/EPICURO studies, respectively. Imputation of missing genotypes was performed using IMPUTE v2 [21], and genotypes of SBC/EPICURO controls were pre-phased to produce best-guess haplotypes using SHAPEIT v2 software [22]. For both PanGenEU and EPICURO studies, the 1000 G (Phase 3, v1) reference dataset was used [23].

#### Association analyses

A final set of 317,270 common SNPs (MAF > 0.05) that passed quality control in both studies and showed comparable MAF across genotyping platforms was used. We ensured the inclusion of the 40 variants previously associated with PC risk in individuals of Caucasian origin compiled in GWAS Catalog [5]. Logistic regression models [24] were computed assuming an additive mode of inheritance for the SNPs, adjusted for age at PC diagnosis or at control recruitment, sex, the area of residence [Northern Europe (Germany and Sweden), European islands (UK and Ireland), and Southern Europe (Italy and Spain)], and the first 5 principal components (PCA) calculated with *prcomp* R function based on the genotypes of 32,651 independent SNPs (J Tyrer, personal communication) to control for potential population substructure.

#### Validation of the novel GWAS hits

To replicate the top 20 associations identified in the Discovery phase, we performed a meta-analysis using risk estimates obtained in previous GWAS from the Pancreatic Cancer Cohort Consortium (PanScan: https://epi.grants.cancer.gov/PanScan/) and the Pancreatic Cancer Case-Control Consortium (PanC4: http://www.panc4.org/), based on 16 cohort and 13 case-control studies, respectively. Details on individual studies, namely PanScan I, PanScan II, PanScan III, and PanC4, have been described elsewhere [6–9]. Genotyping for PanScan studies was performed at the NCI Cancer Genomic Research Laboratory using HumanHap550v3.0, and Human 610-Quad genotyping platforms for PanScan I and II, respectively, and the Illumina Omni series arrays for PanScan III. Genotyping for PanC4 was performed at the Johns Hopkins Center for Inherited Disease Research using the Illumina HumanOmniExpressExome-8v1 array. PanScan I/II datasets were imputed together while PanScan III and PanC4 were each imputed independently using the 1000 G (Phase3, v1) reference dataset [23] and IMPUTE2 [21]. Association models were adjusted for study (PanScan I and II), geographical region (for PanScan III), age, sex, and PCA of population substructure (5 PCA for PanScan I+II, 6 for PanScan III) for PanScan models, and for study, age, sex, and 7 PCA population substructure for PanC4 models. Summary statistics from PanScanI/II, PanScan III, and PanC4 were used for a meta-analysis using a random-effects model based on effect estimates and standard errors with the metafor R package [25].

### 2D approach: Local Moran Index

#### Local Moran’s Index calculation

The LMI was obtained for each SNP considered in the GWAS (*n* = 317,270) using the summary statistics resulting from the association analyses as follows. First, we standardized the OR of each SNP after referring it to the risk-increasing allele (i.e., OR > 1) using the inverse of the normal distribution. Then, we calculated the weight matrix containing the linkage disequilibrium (*r*^2^) as proxy for the distance between each SNP and each of its neighboring SNPs (± 500 kb). SNPs present within this window were matched by MAF to maximize the chance that haplotypes match. Therefore, the LMI for *i*th SNP was calculated as:

$$ {LMI}_i={z}_i\times \sum \frac{z_j\times {r}_{i,j}^2}{\sum {r}_{i,j}^2}, $$

where *LMI*_*i*_ is the LMI value for the *i*th SNP; *z*_*i*_ is the OR value for the *i*th SNP, obtained from the inverse of the normal distribution of ORs for all SNPs; *z*_*j*_ is the OR for the *j*th SNP within the physical distance and MAF-matched defined bounds; and ${r}_{i,j}^2$ is the LD value, measured by *r*^2^, between the *i*th SNP and the *j*th SNP [26].

The LMI score could be estimated for 98.8% of the SNPs in our dataset, as 1.2% of the SNPs were not genotyped in the 1000 G (Phase 3, v1) reference dataset [23] or had a MAF < 1% in the CEU European population (*n* = 85 individuals, phase 1, version 3). We then discarded the SNPs that (1) had a negative LMI, meaning either that surrounding SNPs and target SNP have largely different ORs or that they are in linkage equilibrium and, therefore, do not pertain to the same cluster, or (2) had a positive LMI, i.e., target and surrounding SNPs have similar ORs, but the SNP came from the bottom 50% tail of the distribution of the ordered transformed OR distribution.

To assess the usefulness of the LMI score for SNP prioritization, we ran two benchmarking tests. First, we evaluated whether the GWAS Catalog PC-associated SNPs known to be associated with PC in European populations (GWAS Catalog, *n* = 40 [5]) had a LMI value higher than expected. Then, we assessed how many of the previously reported loci were also identified according to the LMI out of the 30 independent signals of ≥ 1 SNPs. Further details can be found in Additional file 1: Supplementary methods.

### 3D approach: Hi-C pancreas interaction maps and interaction selection

The 3D Hi-C interaction maps for both healthy pancreas tissue [27] and for a pancreatic cancer cell line (PANC-1) were generated using TADbit as previously described [28]. Briefly, Hi-C FASTQ files for 7 replicas of healthy pancreas tissue were downloaded from GEO repository (Accession number: GSE87112; Sequence Read Archive Run IDs: SRR4272011, SRR4272012, SRR4272013, SRR4272014, SRR4272015, SRR4272016, SRR4272017), and for PANC-1 FASTQ, files were available from ENCODE (Accession number: ENCSR440CTR). Merged FASTQ files of the 7 healthy samples and those of PANC-1 were mapped against the human reference genome hg19, parsed and filtered with TADbit to get the final number of valid interacting read pairs (99,074,082 and 287,201,883 valid interaction pairs, respectively). From this set, we built chromosome-wide interaction matrices at 40 kb resolution. The HOMER package [29] was used to detect significant interactions between bins using the –center and --maxDist 2000000 parameters. Using HOMER’s default parameters, the final number of nominally significant (*p* value ≤ 0.001) interactions was 41,833 for the healthy dataset and 357,749 for the PANC-1 dataset. To further filter the interactions, we retained those that passed a Bonferroni corrected threshold < 1 × 10^−5^, resulting in 6761 for the healthy sample (16.2% top interactions from those originally selected by HOMER default parameters). To make it comparable, we also kept the top 16.2% interactions identified in PANC-1, resulting in 57,813 significant interactions.

### Functional in silico analysis

An exhaustive in silico analysis was conducted for associations with *p* values < 1 × 10^−4^ in the PanGenEU GWAS (*N* = 143) and for the top 0.5% loci according to their LMI (*N* = 510) (Additional file 1: Figure S1). Bioinformatics assessments included evidence of functional impact [30–32], annotation in overlapping genes and pathways [31], methylation quantitative trait locus in leukocyte DNA from a subset of the PanGenEU controls (mQTLs), expression QTL (eQTLs) in normal and tumoral pancreas (GTEx and TCGA, respectively) [33, 34], annotation in PC-associated long non-coding RNA (lncRNAs) [35], protein quantitative trait locus analysis in plasma (pQTLs) [36], overlap with regulatory chromatin marks in pancreatic tissue obtained from ENCODE [37], association with relevant human diseases [38], and annotation in differentially open chromatin regions (DORs) in human pancreatic cells [39]. We also investigated whether prioritized variants had been previously associated with PC comorbidities or other types of cancers [5].

We also computed the *credible sets* (calculated following the procedure in [40]; code at https://github.com/hailianghuang/FM-summary), with an *r*^2^ > 0.1, physical distance ± 500 kb, and up to a posterior probability of 0.99 for the variants prioritized by the 1D (*N* = 143 SNPs) and the 2D (510 SNPs) approaches within a 1-Mb window.

In addition to the in silico functional analyses at the variant level, we conducted enrichment analyses at the gene level using the FUMAGWAS web tool [38] and investigated whether our prioritized set of genes appeared altered at the tumor level in a collection of pancreatic tumor samples [41]. Methodological details of all bioinformatics analyses conducted are described in detail in Additional file 1: Supplementary methods.

## Results

### 1D approach: PanGenEU GWAS—single marker association analyses

We performed a GWAS including data from 1317 patients diagnosed with PC (cases) and 1616 control individuals from European countries. In addition to the genotyped SNPs that passed the quality control, we considered the imputed genotypes for previously reported PC-associated hits not included in the OncoArray-500K (19 SNPs with info score ≥ 0.91). In all, 317,270 SNPs were tested (Additional file 1: Figure S2) with little evidence of genomic inflation (Additional file 1: Figure S3).

#### Replication of previously reported GWAS hits

Of the 40 previously GWAS-discovered variants associated with PC risk in European ancestry populations [5], 17 (42.5%) were replicated with nominal *p* values < 0.05. For all 17, the associations were in the same direction as in the primary reports (Additional file 1: Table S2). Among them, we replicated *NR5A2*-rs2816938 and *NR5A2*-rs3790844, a gene for which extensive experimental evidence supporting a role in PC has been acquired. We also observed significant associations for seven variants tagging *NR5A2* previously reported in the literature [7–10, 42]. Replicated GWAS hits included *LINC00673*-rs7214041, reported to be in complete LD with *LINC00673-*rs11655237 [11], previously shown to be a PC-associated variant [9]. At the GWAS significance level, we also replicated *TERT*-rs2736098 [8, 11].

#### The top 20 PanGenEU GWAS hits: validation in independent populations

The risk estimates of the top 20 variants in the PanGenEU GWAS were included in a meta-analysis with those derived from PanScanI+II, PanScan III, and PanC4 consortia GWAS, representing a total of 10,357 cases and 14,112 controls (Additional file 1: Table S3). PanGenEU identified a new variant in *NR5A2* associated with PC (*NR5A2*-rs3790840, metaOR = 1.23, *p* value = 5.91 × 10^−6^) which is in moderate LD with *NR5A2*-rs4465241 (*r*^2^ = 0.45, metaOR = 0.81, *p* value = 3.27 × 10^−10^) and had previously been reported in a GWAS pathway analysis [42]. *NR5A2*-rs3790840 remained significant (*p* value < 0.05) when conditioned on *NR5A2-*rs4465241, on *NR5A2-*rs3790844 plus *NR5A2-*rs2816938, and even on the 13 *NR5A2* GWAS hits reported in the literature, indicating that *NR5A2*-rs3790840 is a new, distinct, PC risk signal. The SKAT-O [43] (seqMeta R package: https://rdrr.io/cran/seqMeta/man/skatOMeta.html), a gene-based analysis considering all significant *NR5A2* hits plus *NR5A2*-rs3790840, yielded a significant association (*p* value = 8.9 × 10^−4^). Furthermore, in a case-only analysis conducted within the PanGenEU study, the overall *NR5A2* variation was associated with diabetes (*p* value = 6.0 × 10^−3^), suggesting an interaction between both factors in relation to PC risk.

While not replicated in the meta-analysis or not in the top 20 SNPs, other variants of interest identified by the 1D approach are located in *SETDB1*, *FAM63A*, *SCTR*, *SEC63*, *CASC8*, and *RPH3AL* loci (Table 1). Their potential functionality is commented below.

**Table 1:** Novel pancreatic cancer genetic susceptibility hits prioritized by approaches 1D, 2D, and 3D, as well as by in silico functional analyses

Genomic location	No. of prioritized novel SNPs	Nearest gene	Selection approach	Relevance in pancreas physiology or carcinogenesis
**1**:150902203, 150974311	2	*SETDB1*, *FAM63A/MINDY1*	1D, 2D	Histone methyltransferase that cooperates in the development of PC; lysine 48 deubiquitinase
**1**:200016460	1	*NR5A2*	1D, 2D	Transcription factor required for acinar differentiation and PC susceptibility gene
**2**:120278171	1	*SCTR*	1D	Secretin receptor expressed in ductal cells
**6**:108238917	1	*SEC63*	1D	ER protein involved in ER stress response
**6**:117150008	1	*GPRC6A*	2D	Disease-causal variant (CADD score = 35)
**6**:117196211	1	*RFX6*	2D	Transcription factor involved in pancreatic development and adult endocrine cell function
**8**:128302062-128494384	27	*CASC8*	1D, 2D	LncRNA cancer associated susceptibility gene
**9**:21967751-21995300	6	*CDKN2A*	2D	Tumor suppressor mutated in > 95% of PC; also involved in familial PC
**9**:6772101, 6785243, 6831637	3	*KDM4C*	2D	Highly expressed in PC
**9**:94601093, 94603970	2	*ROR2*	2D	Wnt pathway (activated in PC)
**11**:60197299	1	*MS4A5*	2D	Disease-causal variant (CADD score = 24.4)
**13**:46446427, 46405544, 46471859-46511859	2/CIR	*SIAH3*	2D, 3D	E3 ubiquitin ligase
**16**:67387817, 67397580	2	*LRRC36*	2D	Disease-causal variant (CADD score = 24.4)
**16**:75263661, 75295639, 75201482-75241482	2/CIR	*BCAR1/CTRB2*	2D, 3D	CTRB2 is chymotrypsinogen 2, a major pancreatic protease; associated with chronic pancreatitis and PC
**17**:143542	1	*RPH3AL*	1D	Regulatory variant; rabphilin 3a is involved in exocytosis in acinar cells
**22**:28476910-29165195	CIR	*MN1*	3D	No pancreas-related function has been discovered so far
**22**:28602352-28642352	CIR	*XBP1*	3D	Major regulator of the ER stress and unfolded protein responses in acinar cells

*PC* pancreatic cancer, *CIR* chromatin interacting region

### 2D approach: genomic spatial integration

We scaled up from the single-SNP (1D) to the genomic region (2D) association analysis by considering both genomic distance (LD) between variants and the magnitude of the association (OR) with the variants. We calculated a LMI score and selected those SNPs with positive LMI or within the top 50% of OR values resulting in a final set of 102,146 SNPs. The LMI scores and *p* values for these variants showed a direct correlation (Spearman *r* = 0.62; *p* value *=* 2.2 × 10^−16^, Additional file 1: Figure S4). To assess the versatility of LMI, we ran two benchmarks based on the MAFs and the ORs. Out of the 30 PC independent signals (*r*^2^ < 0.2) derived from the GWAS Catalog, 22 were present in our 102,146 selected set. The observed median rank position for the 22 PC signals in this list was 22,640, an average position significantly higher than that of 10,000 randomly selected sets of the same size (one tail *p* value = 0.0013) (Additional file 1: Figure S5). Moreover, out of the PC genomic loci, LMI was able to capture those reported by at least two studies (21 out of 30 PC independent genomic loci).

An LMI-enriched variant set was generated by selecting the top 0.5% of SNPs according to their LMI scores (LMI ≥ 5.1071) resulting in 510 SNPs, which included 29 out of the 143 SNPs prioritized by the 1D approach (Additional file 2: Table S4). We compared the MAF of the independent SNPs (*r*^2^ < 0.2) (see Additional file 1: Supplementary methods) prioritized by the 1D approach (*N* = 97/143) against the top 97 independent variants, out of 196 independent signals for the 510 SNPs selected by LMI. Notably, the LMI-identified SNPs had a lower MAF than GWAS-identified variants: 0.07 (SD = 0.03) vs. 0.24 (SD = 0.13) (Wilcoxon statistic *p* value < 2.2 × 10^−16^) (Additional file 1: Figure S6). In line with this observation, the average OR for the LMI-based SNPs was significantly higher than that for the GWAS-based SNPs (1.46 vs. 1.32, respectively, Wilcoxon statistic *p* value *=* 1.63 × 10^−10^).

The Manhattan plot of the LMI score across the genome displays the hits identified through this approach (Additional file 1: Figure S7). Among the 0.5% top LMI prioritized variants (*N* = 510), there were 8 SNPs in *NR5A2*, including the novel PanGenEU GWAS identified variant (rs3790840). All of them showed a high LMI score (> 6.859) what further endorses this approach. Other variants of interest identified by the 2D approach are in *SETDB1*, *FAM63A/MINDY1*, *GPRC6A*, *RFX6*, *CASC8*, *CDKN2A*, *KDM4C*, *ROR2*, *MS4A5*, *SIAH3*, *LRRC36*, and *CTRB2/BCAR1* loci (Table 1). Their potential functionality is discussed below.

A total of 199 *credible sets* were identified among the 510 LMI-based SNP. Of them, 118 (60%) contained the SNP with the lowest *p* value in the region. Moreover, we observed an enrichment of SNPs with low *p* values in the 1-Mb region for the LMI-based SNP set that was even higher among the 118 *credible sets* (Additional file 1: Figure S8).

### 3D approach: genomic interaction analysis

To gain further insight into the biological function of the 624 candidate SNPs prioritized using the 1D and 2D approaches, and to identify additional PC genetic susceptibility loci, we focused on a set of 6761 significant chromatin interactions (*p* values ≤ 1 × 10^−5^) identified using Hi-C interaction pancreatic tissue maps at 40 Kb resolution [27]. Throughout the rest of the text, we will refer to the chromatin interaction component containing the prioritized SNP as “bait” and to its interacting region as “target.” In total, 54 target loci overlapping with 37 genes interacted with bait regions harboring 76/624 (12.1%) SNPs (Additional file 3: Table S5).

**Fig. 2:**
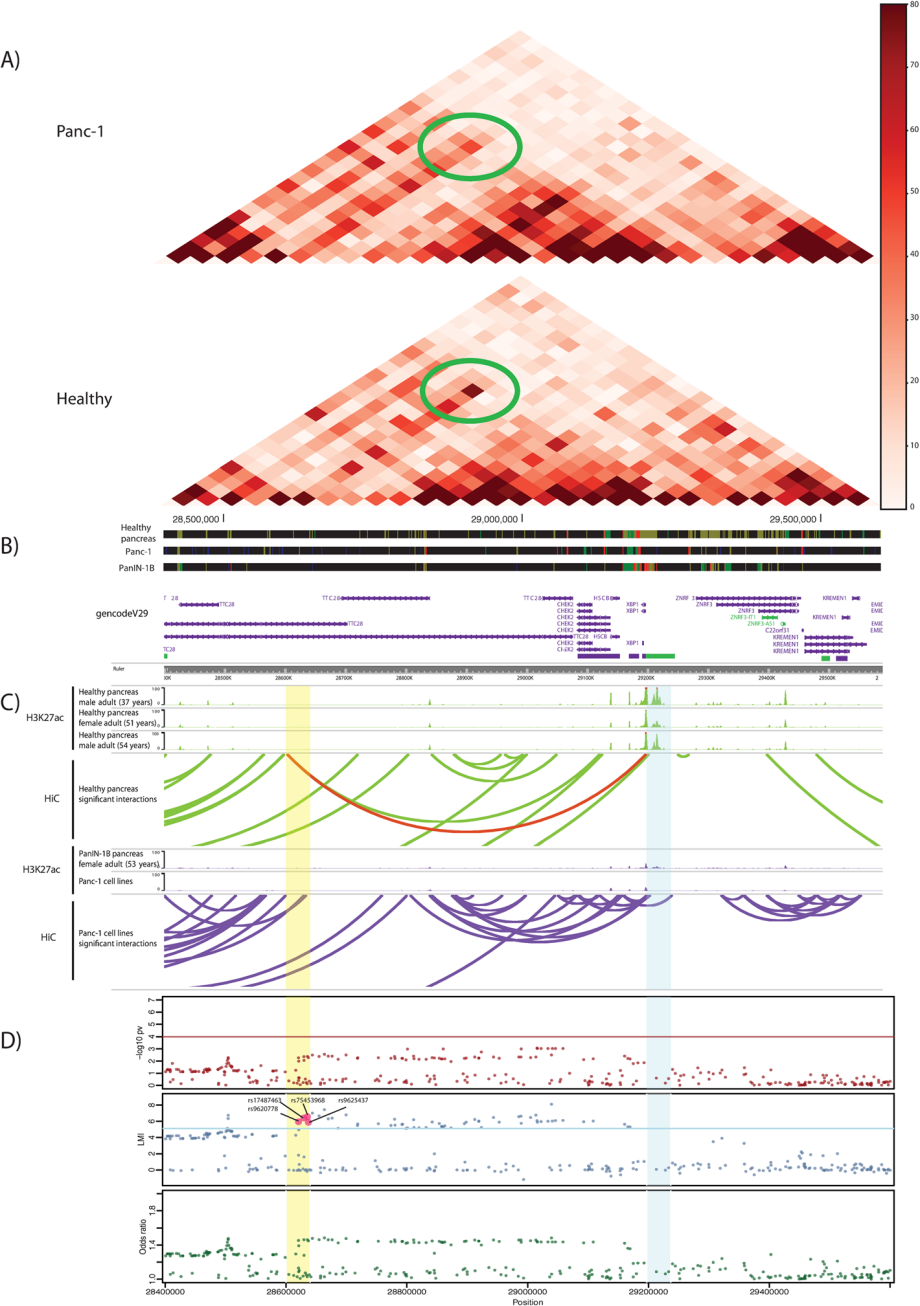
Three-dimensional genome organization in healthy and PANC-1 cells and association results corresponding to the genomic region around *XBP1* using the standard GWAS and 2D approaches. **a** Coverage-normalized Hi-C maps of healthy samples and PANC-1 cells at 40 Kb resolution. Green ellipses highlight the interaction between the region harboring four Local Moran’s Index (LMI)-selected SNPs and the *XBP1* promoter. **b** Tracks of the ChromHMM Chromatin for 8 states in healthy pancreas, PANC-1 cells, and a Pancreatic Intraepithelial Neoplasia 1B. Promoters are colored in light purple, strong enhancers in dark green, and weak enhancers in yellow. Note that the strong enhancer in the target region is lost in the PANC-1 and PanIN-1B samples, compared to the healthy samples. **c** UCSC tracks of H3K27ac, an enhancer-associated mark, and arcs linking significant interactions called by HOMER. Interactions in healthy pancreas samples are in green and those in PANC-1 and in the PanIN-1B sample are in purple. Red arc represents the interaction between LMI-prioritized SNPs and the *XBP1* promoter (highlighted region in Hi-C map in **a**). **d** Scatterplots of SNPs in region chr22:28,400,000-29,600,000 (hg19) and their –log10 (*p* value), LMI, and odds ratio. Bait and target chromatin interaction regions are highlighted in yellow and blue, respectively

As a proof of concept of the utility of the 3D approach to identify novel PC genetic susceptibility loci, we highlight a target region (22:29,197,371-29,237,371 bp, *p* value = 1.3 × 10^−9^) interacting with an intronic region of *TTC28* (bait: 22:28,602,352-28,642,352 bp) that includes four LMI-selected SNPs (rs9620778, rs9625437, rs17487463, and rs75453968, all in high LD, *r*^2^ > 0.95, in CEU population) (Fig. 2). Other loci of interest identified by the 3D approach are in *SIAH3*, *CTRB2*, and *MN1* loci (Table 1). Their potential functionality is commented below.

### Functional in silico validation

We performed a systematic and exhaustive in silico functional analysis of SNPs prioritized by GWAS (*N* = 143) and LMI (*N* = 510) at the variant, gene, and pathway levels (Fig. 1 and Additional file 1: Figure S1).

#### Assessment of potential functionality of the variants

The evidence for potential functionality of the most relevant SNPs for each of the approaches used is reviewed here and summarized in Table 1, Additional file 1: Supplementary methods, and Additional file 2: Table S4 and Additional file 4: Table S6.

**Fig. 3:**
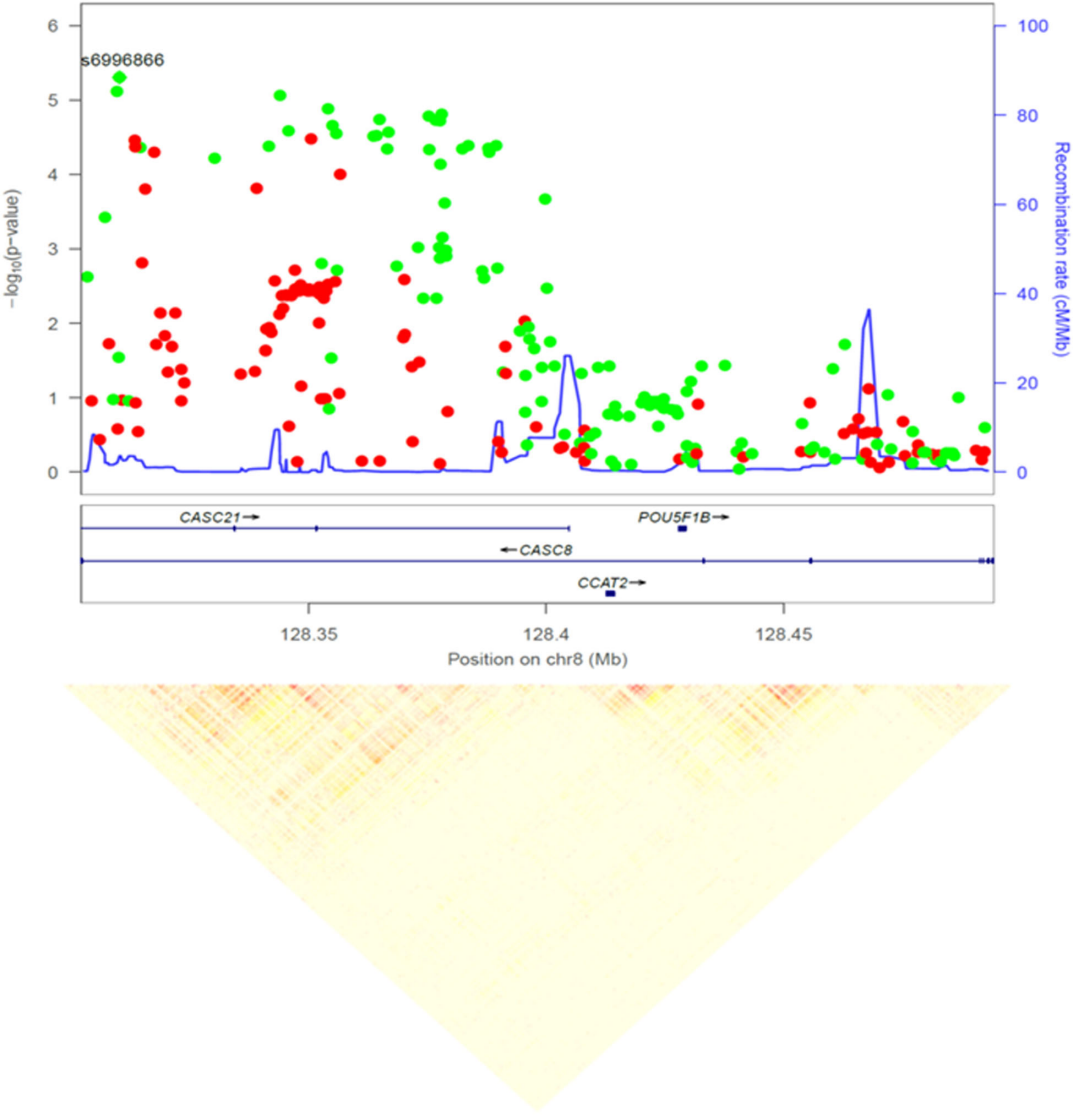
Zoom plot of the 8q24.21 *CASC8* (cancer Susceptibility 8) region and linkage disequilibrium pattern of the PanGenEU GWAS prioritized variants. Red and green points indicate OR < 1 and OR > 1, respectively

Among the 143 variants prioritized in the 1D approach, we highlight those in *CASC8* (8q24.21) (Fig. 3): 27 variants with *p* values < 1 × 10^−4^ organized in four LD-blocks, 9 of which were also captured in the 2D approach. The *CASC8* locus is amplified in 5% of PC and codes for a non-protein coding RNA overexpressed in tumor vs. normal pancreatic tissue (Log2FC = 1.25, *p* value = 2.29 × 10^−56^). *CASC8* also overlaps with a PC-associated lncRNA [35], suggesting that genetic variants in *CASC8* may contribute to the transcriptional program of pancreatic tumor cells. All *CASC8* variants were also associated with differential leukocyte methylation (mQTL) of *RP11-382A18.1-*cg25220992 in our PanGenEU population sample. Moreover, 20 of them were associated with differential methylation of cg03314633, also in *RP11-382A18.1*. Twenty-three of the variants overlapped with at least one histone mark in either endocrine or exocrine pancreatic tissue. Alterations in *CASC8* significantly co-occur with alterations in *TG* (adjusted *p* values *<* 0.001), also associated with PC in our GWAS, which is located downstream.

Three of the variants prioritized for in silico analysis in the 1D approach (but not in the 2D approach) are located in genes involved in pancreatic function: rs1220684 is in *SEC63*, coding for a protein involved in endoplasmic reticulum (ER) function and ER stress response [44]; rs7212943, a putative regulatory variant, is in *NOC2/RPH3AL*, a gene involved in exocytosis in exocrine and endocrine cells [45]; and rs4383344 is in *SCTR*, which encodes for the secretin receptor, selectively expressed in ductal cells, involved in the regulation of bicarbonate, electrolyte, and volume secretion. Interestingly, secretin regulation is affected by *Helicobacter pylori* which has been suggested as a PC risk factor [46]. High expression of *SCTR* has also been reported in PC [47].

Two variants in high LD (*r*^2^ = 0.92) and potentially relevant at the functional level are in 1q21.3 (*SETDB1*-rs17661062 and *FAM63A-*rs59942146). *SETDB1* has recently been reported to be required for formation of PC in mice by inhibiting p53-mediated apoptosis [48], and *FAM63A/MINDY1* has been found to interact significantly with diabetes (duration ≥ 3 years) in a meta-analysis on PC risk conducted within the PanC4 and PanScan consortia [49]. Interestingly, these two variants were also associated with an increased methylation of the cg17724175 in *MCL1*. High mRNA expression of this gene has been associated with poor survival [50], and Mcl-1 has been explored to selectively radiosensitize PC cells [51]. Importantly, these two variants were also the top two prioritized by the 2D approach with a LMI score > 16.87 (Additional file 2: Table S4).

**Fig. 4:**
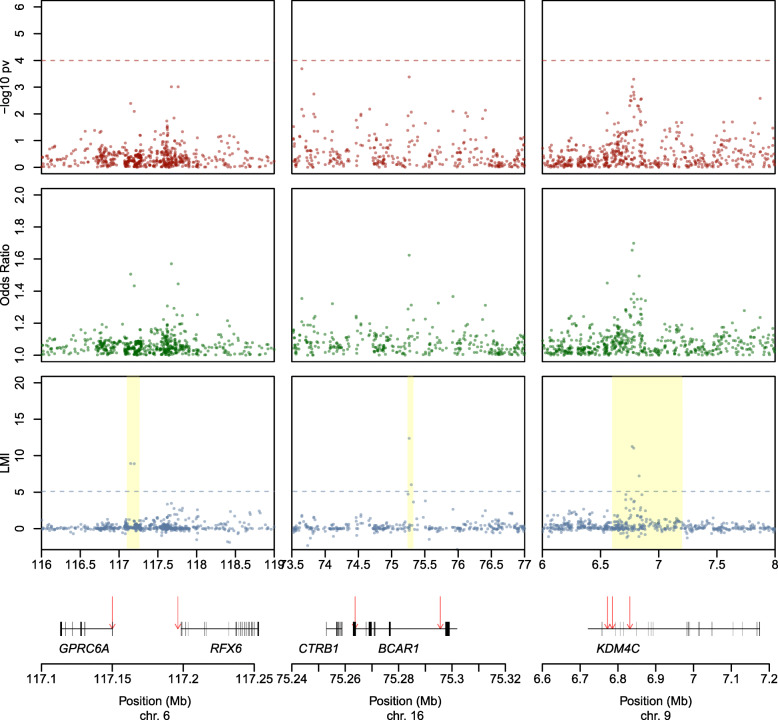
Scatterplots of the –log10 *p* values, Local Moran’s Index (LMI) values, and odds ratios (OR) for three genomic regions prioritized based on their LMI value. Highlighted regions show the hits identified in the 2D, but not in the 1D approach

Using the 2D approach, we prioritized several other regions with potential functional relevance (Table 1, Fig. 4, Additional file 1: Figure S7, Additional file 2: Table S4). In chromosome 6, we identified rs6907580 (LMI = 8.93), a well-characterized stop-gain—and likely disease-causal variant (CADD score = 35)—in exon 1 of *GPRC6A* (*G protein-coupled receptor family C group 6 member A*). *GPRC6A* is expressed in pancreatic acinar, ductal, and β-cells; it participates in endocrine metabolism [52]; and it has been involved in pancreatitis using mouse models [53]. Downstream in the same region, LMI approach also identified rs17078438 (LMI = 8.90) in *RFX6*, a pancreas-specific gene involved in pancreatic development [39].

Other potentially functional SNPs relevant to PC and prioritized in the 2D approach comprised 6 SNPs (LMI ≤ 5.60) in the vicinities of *CDKN2A/p16*, a gene that is almost universally inactivated in PC [54] and that is mutated in some hereditary forms of PC [55, 56], with three variants (LMI ≤ 5.48) in *CDKN2A-AS1* and two (LMI ≤ 5.88) in *CDKN2B/p15*, other important cell-cycle regulators; three variants in *KDM4C* (LMI ≤ 11.27), a Lys demethylase 4C highly expressed in PC [57]; and two SNPs tagging *ROR2* (LMI ≤ 5.57), a member of the *Wnt* pathway that plays a relevant role in PC [58].

Another region, in chromosome 16, comprises *BCAR1-*rs7190458, a variant with a relevant role in PC [59] reported in two previous GWAS [8, 11], as well as a novel SNP (rs13337397, LMI = 6.03) located in the first exon of *BCAR1.* Both SNPs are in low LD (*r*^2^ = 0.36). This second SNP is intergenic to *CTRB2* and *BCAR1* (Table 1, Fig. 4, Additional file 1: Figure S7, Additional file 2: Table S4). While *BCAR1* is ubiquitous, *CTRB2* code for chymotrypsinogens B2, a protease expressed exclusively in the exocrine pancreas; genetic variation therein has been previously associated with alcoholic pancreatitis [60] and type 2 diabetes [61, 62]. The expression of that gene is reduced in tumors vs. normal tissue [63]. Our finding using the 3D-approach further supports that this locus harbors genetic variation of relevance to PC risk.

According to their deleteriousness CADD score [32], we highlight two variants in coding transcripts: *MS4A5*-rs34169848 in chr11:60,197,299 (CADD score = 24.4) and *LRRC36*-rs8052655 in chr16:67,409,180 (CADD score = 24.4). CADD scores of such magnitude are likely to correspond to disease-causal variants [64].

The 3D approach highlighted *XBP1* as a target region. As said, this target region including the *XBP1* promoter interacts with four of the LMI-selected SNPs (Fig. 2) that are in moderate LD with rs16986177. The alternative allele (T) of this SNP is associated with a decreased expression of *XBP1* in normal pancreas in GTEx (− 0.19, *p* value = 1.3 × 10^−4^) and with an increased risk of PC in our GWAS (OR = 1.28, *p* value = 8.71 × 10^−3^). Expression of *XBP1* is reduced in PC samples from TCGA, compared to normal pancreas samples from GTEx (Log2FC = − 1.561, *p* value = 1.72 × 10^−34^). Chip-Seq data of all pancreatic samples available in ENCODE, as well as PANC-1 pancreatic cancer cells (see the “Methods” section), allowed us to find that, in comparison to normal pancreas, the H3K27Ac mark present in the *XBP1* promoter is completely lost in PANC-1 cells and is reduced in a sample of a Pancreatic Intraepithelial Neoplasia 1B, a PC precursor (Fig. 2). To further characterize the bait and promoter regions upstream of *XBP1*, we ran eight chromatin states using ChromHMM [65] (Additional file 1: Supplementary methods). We observed a clear loss of enhancers/weak promoters in the corresponding target regions in the precursor lesions and in PANC-1 cells. This loss of activity is in line with the observation that *XBP1* expression is reduced in cancer. Moreover, small enhancers are also lost in the bait region of the aforementioned samples. The 3D maps for this region revealed loss of 3D contact in PANC-1 cells (Fig. 2).

#### Gene set enrichment analyses (GSEA)

**Fig. 5:**
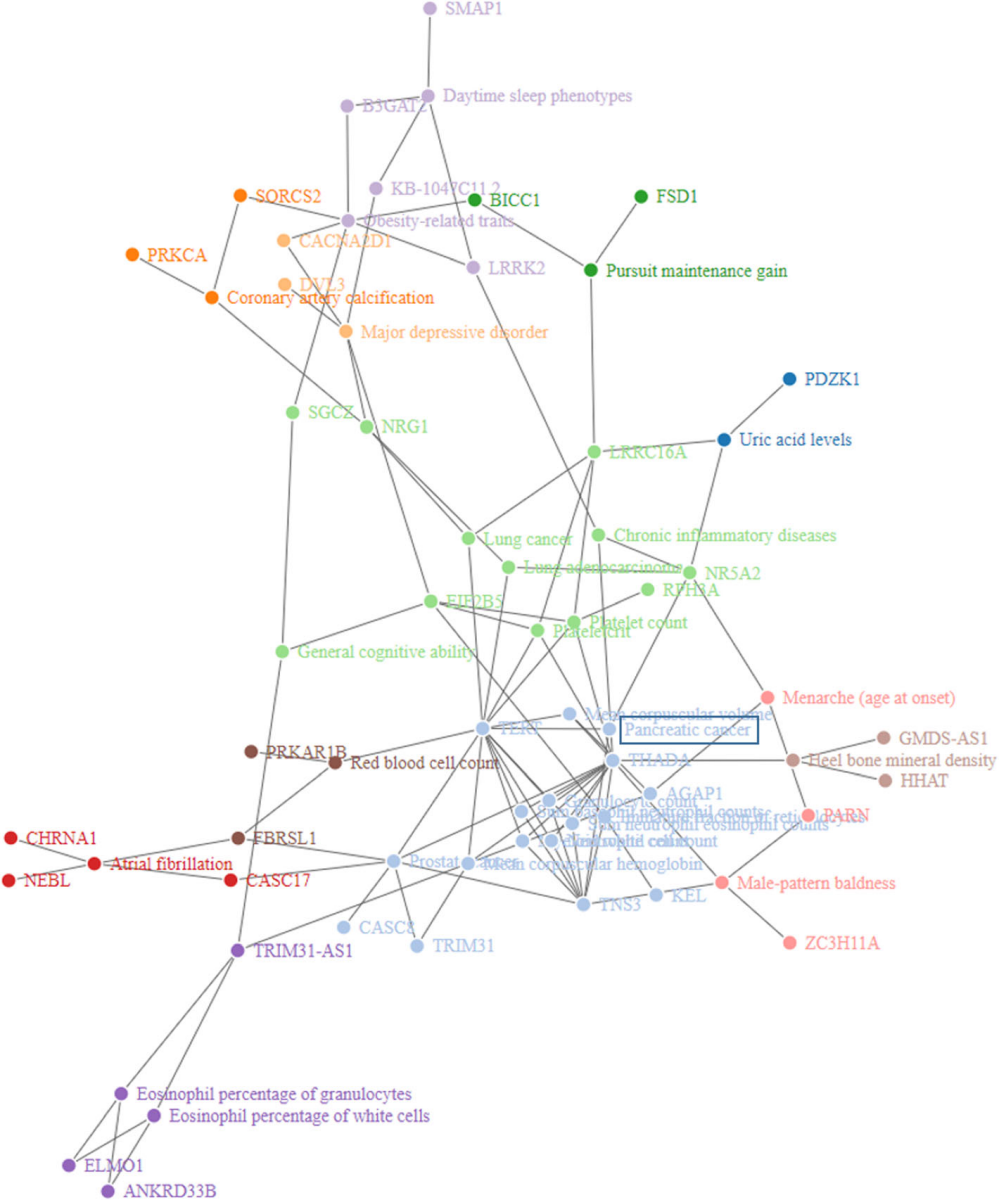
Network of traits in the GWAS Catalog enriched with the genes prioritized in the 1D approach of PanGenEU GWAS. Twelve densely connected subgraphs identified via random walks are displayed in different colors

We performed GSEA of the genes harboring the SNPs prioritized using the 1D and 2D approaches. Six chromosomal regions were significantly enriched among the 81 genes harboring the 143 prioritized SNPs in the 1D approach (Additional file 5: Table S7). GSEA for the gene-trait associations reported in the GWAS Catalog yielded 29 enriched traits (Additional file 5: Table S7). The most relevant GWAS traits significantly enriched were “Pancreatic cancer,” “Lung cancer,” “Prostate cancer,” “Uric acid levels,” “Obesity-related traits,” and “Major depressive disorder.” We also performed a network analysis to visualize the relationships between the enriched GWAS traits and the prioritized genes using the *igraph* R package [66]. Twelve densely connected subgraphs were identified via random walks (Fig. 5). Interestingly, “pancreatic cancer” and “uric acid levels” GWAS traits were connected through *NR5A2*, which is also linked to “chronic inflammatory diseases” and “lung carcinoma” traits. *NR5A2* is an important regulator of pancreatic differentiation and inflammation in the pancreas [67].

GSEA of the genes harboring the variants included in the *credible sets* corresponding to the 2D approach revealed enrichment in “Pancreatic cancer” as well as other GWAS traits related to PC risk factors, including alcoholic chronic pancreatitis, type 2 diabetes, body mass index and waist-to-hip ratio adjusted for body mass index, and HDL cholesterol (Additional file 6: Table S8). These findings lend support to the validity of the 2D approach as a tool to identify disease-relevant genetic variants.

#### Pathway enrichment analysis

The genes prioritized in the 1D approach were significantly enriched in 112 Gene Ontology Biological Function (GO:BP) terms (adjusted *p* values < 0.05, with minimum of three genes overlapping), 7 Cellular Component GO terms (GO:CC), and 11 Molecular Function (GO:MF) terms (Additional file 5: Table S7). Importantly, GO terms relevant to exocrine pancreatic function were overrepresented. Three KEGG pathways were significantly enriched with ≥ 2 genes from our prioritized set, including “Glycosaminoglycan biosynthesis heparan sulfate” (*adj*-*p* = 3.86 × 10^−3^), “ERBB signaling pathway” (*adj*-*p* = 3.73 × 10^−2^), and “Melanogenesis” (*adj*-*p* = 3.73 × 10^−2^) (Additional file 5: Table S7). Pathways enriched with the genes prioritized in the 2D approach included GO terms related to the nervous system and G protein-coupled receptor signaling. Interestingly, one of the hallmarks of PC is perineural invasion. Because the standard databases generally lack pathways related to acinar pancreatic function, we generated several curated gene sets and assessed their enrichment among the SNPs/genes prioritized in the 1D and 2D approaches. We found an overrepresentation of LMI genes in a signature including transcription factors differentially expressed in normal pancreas (GTEx). This signature was also enriched with genes prioritized in the other two approaches used in our study (including 11 overlapping genes: *SETDB1*, *LHX4*, *NR5A2*, *ZBED6*, *ELK4*, *SIM1*, *RFX6*, *KLF14*, *ZNF32*, *ZNF133*, and *XBP1*).

In summary, the in silico functional analysis revealed a remarkable enrichment of pathways related to the function of acinar and ductal cells, including SNPs associated with novel genes in these pathways.

## Discussion

To overcome some of the limitations of standard GWAS analyses, we have expanded the scope of genomic studies of PC susceptibility to include novel approaches that build on spatial genome autocorrelations of LMI and 3D chromatin contacts. An in-depth in silico functional analysis leveraging available genomic information from public databases allowed us to prioritize new candidate variants with strong biological plausibility in well-established (i.e., *NR5A2*) as well as in novel (i.e., *XBP1*) genes playing a key role in acinar function (Table 1). We have thus reached a novel landscape on the inherited basis of PC and have paved the way to the application of a similar strategy to any other human disease or interest.

This is the first PC GWAS involving an exclusively Europe-based population sample. Of the previously reported European ancestry population GWAS hits, 42.5% were replicated, supporting the methodological soundness of the study. The lack of replication of other PC GWAS hits may be explained by variation in the MAFs of the SNPs among Europeans, population heterogeneity, differences in the genotyping platform used, and differences in calling methods applied, among others. This result emphasizes that statistical significance for GWAS-SNPs is largely dependent on MAF and the statistical power of the study, highlighting this as a major limitation of classical GWAS analyses.

We applied the LMI (2D approach) for the first time in the genomics field. LMI captured a new dimension of signals independent from MAF and the statistical power of the study (Additional file 1: Figure S6). The benchmarking tests evidenced that LMI prioritizes SNPs on the basis of OR that were largely present in *credible sets* (Additional file 1: Figure S8). We replicated 6.4% of the previous reported GWAS Catalog signals for PC in European populations by considering the top 0.5% LMI variants, a LMI threshold that is overly conservative, given that many of the GWAS Catalog-replicated signals have lower LMI than the cutoff value we selected. The ability of LMI to prioritize low MAF SNPs, unlike the GWAS approach, may also explain the low replicability rate. LMI helps to identify signals within genomic regions by scoring lower those regions that do not maintain LD structure.

The 3D genomic approach identified a highly potential important chromatin interacting region in *XBP1*. This is a potential candidate detected through a previously uncharacterized “bait” SNP. These findings are particularly important considering the overwhelming evidence of a major role of ER stress and unfolded protein responses in acinar function—two highly relevant processes to acinar homeostasis due to their high protein-producing capacity of these cells—and it plays an important role in pancreatic regeneration [68]. In addition, genetic mouse models have unequivocally shown that *Xbp1* is required for acinar homeostasis and pancreatic ductal adenocarcinoma, the most common form of PC, can be initiated from acinar cells [69]. Overall, these analyses indicate that the SNPs interacting in 3D space with the *XBP1* promoter could contribute to the differential expression of the gene associated with malignant transformation. These findings provide proof of concept that 3D genomics can contribute to identify further susceptibility loci and to decipher the biological relevance of orphan SNPs. Similar results were found with other LMI-selected SNPs associated with their target genes only by detecting significant spatial interactions between them (Additional File 3: Table S5).

To shed light into the functionality of the newly identified variants, we applied novel post-GWAS approaches to interrogate several databases at the SNP, gene, and pathway levels. We found sound evidence pointing to the functional relevance of several variants prioritized by the 1D and 2D approaches (Additional files 2 and 4: Tables S4 and S6, respectively, and Additional file 1: Supplemental methods). The importance of the multi-hit *CASC8* region (8q24.21) is further supported by in silico functional analyses as well as by its previous associations with PC at the gene level [35]. In particular, 12/27 SNPs identified in *CASC8* were annotated as regulatory variants. None of the *CASC8* hits were in LD with *CASC11*-rs180204, a GWAS hit previously associated with PC risk, which is ~ 205 Kb downstream [10]. *CASC8*-rs283705 and *CASC8*-rs2837237 (*r*^2^ = 0.68) are likely to be functional with a score of 2b in RegulomeDB (TF binding + any motif + DNase Footprint + DNase peak). *CASC8-*rs1562430, in high LD (*r*^2^ > 0.85) with 18 *CASC8* prioritized variants, has been previously associated with other cancers (breast, colorectal, and stomach) [70]. None of the prostate cancer-associated SNPs in *CASC8* overlapped with the 27 identified variants in our study. The fact that this gene has not been reported previously in other PC GWAS could be due to the different genetic background of the study populations or to an overrepresentation of the variants tagging *CASC8* in the Oncoarray platform used here.

In addition to confirming SNPs in *TERT*, we found strong evidence for the participation of novel susceptibility genes in telomere biology (*PARN*) and in the post-transcriptional regulation of gene expression (*PRKCA* and *EIF2B5*) (Additional File 1: Supplemental methods). Our study also expands the landscape of variants and genes involved in exocrine biology, including *SEC63*, *NOC2*/*RPH3AL*, and *SCRT* whose products participate in acinar function and possibly in acinar-ductal metaplasia, a PC pre-neoplastic lesion [71].

KEGG pathway enrichment analysis further validated our results being involved in important pathways for PC, including “Glycosaminoglycan biosynthesis heparan sulfate” and “ERBB signaling pathway.” Heparan sulfate (HS) is formed by unbranched chains of disaccharide repeats which play roles in cancer initiation and progression [72]. Interestingly, the expression of HS proteoglycans increases in PC [73] and related molecules, such as hyaluronic acid, are important therapeutic targets in PC [74, 75]. ERBB signaling is important both in PC initiation and as a therapeutic target [76].

The enrichment analysis indicates that urate levels, depression, and body mass index—three GWAS traits previously reported to be associated with PC risk—were enriched in our prioritized gene set. Urate levels have been associated with both PC risk and prognosis [77, 78]. In addition, patients with lower relative levels of kynurenic acid have more depression symptoms [79]. PC is one of the cancers with the highest occurrence of depression preceding its diagnosis [80]. Furthermore, body mass index has been previously associated with PC risk in diverse populations [81–83] and it has been suggested that increasing PC incidence may be partially attributed to the obesity epidemic. Insulin resistance is one of the mechanisms possibly underlying the obesity and PC association, through hyperinsulinemia and inflammation [84].

The post-GWAS approach used has limitations that should be addressed in future studies. For example, our study has a relatively small sample size, some imbalances regarding gender and geographical areas, and the Hi-C maps that we used have limited resolution (40 kb). To account for population imbalances, regression models were adjusted for gender and for country of origin, as well as for first five principal components. The study of a European-only population allows reducing the population heterogeneity not only at the genetic but also at the non-genetic level. This is particularly advantageous given the novel nature of the analysis performed here and the relatively small sample size of our study. More so, LMI is based on both the summary statistics and LD structure; therefore, it was important to test its validity for the first time in a more homogeneous population, with individuals sharing a more consistent LD pattern. It is now warranted to extend this approach to more generalizable multi-ethnic populations.

Our study has many other strengths: a standardized methodology was applied in all participating centers to recruit cases and controls, to collect information, and to obtain and process biosamples; state-of-the-art methodology was used to extend the identification of variants, genes, and pathways involved in PC genetic susceptibility. Most importantly, the combination of GWAS, LMI, and 3D genomics to identify new variants is completely novel and has proven crucial to refine results, reduce the number of false positives, and establish whether borderline GWAS *p* value signals could be true positives. These three strategies, together with an in-depth in silico functional analysis, offer a comprehensive approach to advance the study of PC genetic susceptibility.

## Conclusions

We present a novel multilayered post-GWAS assessment on genetic susceptibility to PC. We showed that the combined use of conventional GWAS (1D) analysis with LMI (2D) and 3D genomic approaches allows enhancing the discovery of novel candidate variants involved in PC. Importantly, several of the new variants are located in genes relevant to the biology and function of acinar and ductal cells.

This multi-step strategy, combined with an in-depth in silico functional analysis, offers a comprehensive approach to advance the study of PC genetic susceptibility and could be applied to other diseases.

## Supplementary Information

**Additional file 1: Table S1.** Characteristics of the study populations. **Table S2.** Replication of the SNPs reported as associated with pancreatic cancer risk in European population and published in GWAS Catalog. **Table S3.** Validated variants (at the nominal *p*-value), in PanScan and PanC4 populations, among the top 20 SNPs identified in the PanGenEU GWAS study. **Figure S1.** Functional *in-silico* analysis strategy followed to identify novel genomic regions previously prioritized using the 1D, 2D and 3D approaches. **Figure S2.** GWAS Manhattan plot for the PanGenEU study. The x-axis is the genomic position of each variant and the y-axis is the −log10 *p*-value obtained in the 1D analysis. **Figure S3.** Q-Q plots for pancreatic cancer risk of the association results using the PanGenEU case-control study (S2a) and PanGenEU&EPICURO study populations (S2b). **Figure S4.** Scatterplot of the local Moran’s index (LMI) obtained in the 2D approach and the –log10 *p*-value obtained in the GWAS analysis (1D approach). **Figure S5.** Results of the benchmarking test showing that the median rank position of the LMI values for the 22 pancreatic cancer signals from the GWAS Catalog is significantly higher than 10,000 randomly selected sets of the same size. **Figure S6.** Minor allele frequency (MAF) distributions for the top 97 SNPs identified by LMI (in pink) and by GWAS (in blue). **Figure S7.** LMI Manhattan plot for the PanGenEU study. The x-axis is the genomic position of each variant and the y-axis is the LMI value obtained in the 2D analysis. **Figure S8.** Q-Q plots show significant enrichment of SNPs with low *p*-values in the variants prioritized in the 2D-approach (S8a), and in the credible sets derived from them (S8b). **Figure S9.** Complementary NETWORK (in blue our input KEGG pathways; in green, the complementary pathways interconnected with them) obtained with Pathway-connector webtool.

**Additional file 2: Table S4.** List of the 510 SNPs prioritized according to their Local Moran Index (LMI).

**Additional file 3: Table S5.** List of the 76 SNPs overlapping with a chromatin interaction region (bait regions) and their 54 targets.

**Additional file 4: Table S6.** Annotation and functional in silico analysis of the 143 prioritized SNPs.

**Additional file 5: Table S7.** Results from the gene enrichment analysis performed with FUMA in the 1D prioritized genes.

**Additional file 6: Table S8.** Results from the gene enrichment analysis performed with FUMA in the 2D prioritized genes.

## Data Availability

GWAS summary statistics generated in this study are available in the GWAS Catalog repository with the accession numbers GCST90011857 (ftp://ftp.ebi.ac.uk/pub/databases/gwas/summary_statistics/GCST90011857) [85] and GCST90011858 (ftp://ftp.ebi.ac.uk/pub/databases/gwas/summary_statistics/GCST90011858) [86]. Code for performing the association analysis and meta-analysis is available at https://github.com/EvangelinaLdM/Multilayered_postGWAS_PanGenEU [24]. Code for calculating LMI is freely available at https://github.com/pollicipes/Local-Moran-Index-1D [26].

## Abbreviations

PC: Pancreatic cancer
GWAS: Genome-wide association study
LMI: Local Moran’s Index
OR: Odds ratio
LD: Linkage disequilibrium
MAF: Minor allele frequency
SBC: Spanish Bladder Cancer
NCI: National Cancer Institute
PCA: Principal components
mQTL: Methylation quantitative trait locus
eQTL: Expression quantitative trait locus
lncRNA: Long non-coding RNA
pQTLs: Protein quantitative trait locus analysis
DORs: Differentially open chromatin regions
CADD: Combined Annotation Dependent Depletion
ER: Endoplasmic reticulum
GO:BP: Gene Ontology Biological Function
GO:CC: Gene Ontology Cellular Component
GO:MF: Gene Ontology Molecular Function
HS: Heparan sulfate
